# Analysis of *KRAS* Mutations of Exon 2 Codons 12 and 13 by SNaPshot Analysis in Comparison to Common DNA Sequencing

**DOI:** 10.1155/2010/789363

**Published:** 2010-12-21

**Authors:** Rica Zinsky, Servet Bölükbas, Holger Bartsch, Joachim Schirren, Annette Fisseler-Eckhoff

**Affiliations:** ^1^Institute of Pathology and Cytology, HSK Wiesbaden, Academic Hospital of University of Mainz, Ludwig-Erhard-Strasse 100, 65199 Wiesbaden, Germany; ^2^Department of Thoracic Surgery, HSK Wiesbaden, Academic Hospital of University of Mainz, Ludwig-Erhard-Strasse 100, 65199 Wiesbaden, Germany

## Abstract

Due to the call for fast *KRAS* mutation status analysis for treatment of patients with monoclonal antibodies for metastatic colorectal cancer, sensitive, economic, and easily feasible methods are required. Under this aspect, the sensitivity and specificity of the SNaPshot analysis in comparison to the commonly used DNA sequencing was checked. We examined *KRAS* mutations in exon 2 codons 12 and 13 with DNA sequencing and SNaPshot analysis in 100 formalin-fixed paraffin-embedded tumor tissue samples of pancreatic carcinoma, colorectal carcinoma, and nonsmall cell lung cancer specimens of the primary tumor or metastases. 40% of these samples demonstrated mutated *KRAS* genes using sequencing and SNaPshot-analysis; additional five samples (45/100) were identified only with the SNaPshot. *KRAS* mutation detection is feasible with the reliable SNaPshot analysis method. The more frequent mutation detection by the SNaPshot analysis shows that this method has a high probability of accuracy in the detection of *KRAS* mutations compared to sequencing.

## 1. Introduction

Colorectal cancer, nonsmall cell lung cancer (NSCLC), and pancreatic carcinomas are three of the most frequent causes of cancer mortality in the world.

Established therapies are targeted on the epidermal growth factor receptor (EGFR), such as Panitumumab [1–4] and Cetuximab [5–8] (two monoclonal antibodies) treatment for metastatic colorectal carcinoma, Gefitinib for NSCLC, or Erlotinib for NSCLC and for pancreatic carcinoma. Treatment success using Cetuximab and Panitumumab for treatment of metastatic colorectal cancer depends on a nonmutated *KRAS* gene; the treatment is ineffective if the *KRAS* gene has any mutations [1, 5, 6, 8], which leads to an activated G-protein even in absence of the ligand, like the epidermal growth factor [9, 10]. This results in further malignant proliferation of the cells despite treatment. 

Two codons in the *KRAS *gene are mainly known to generate alternated proteins that are constitutively activated without the signal of a ligand bound to the EGFR. This are the codons 12 and 13 in the exon 2 of the *KRAS* gene [9, 10]. Both codons encode the amino acid glycine in the wild type protein. Replacement of one of the first two bases leads in both codons to an amino acid exchange in the KRAS protein, resulting in resistance of the tumor to the above-described treatment. 

Replacement of glycine leads to resistance of the GAPs, which are proteins causing the hydrolysis of KRAS bound GTP to GDP. The inability of GAPs to effect the GTP hydrolysis in mutated KRAS leads to a constitutive active protein [10].

Up to now *KRAS* mutation analysis is mainly performed by DNA sequencing [5] and commercialised test systems by DNA-DNA hybridisation (e.g., Chipron, Berlin, Germany), pyrosequencing [11], or the Therascreen: K-RAS Mutation Kit from Qiagen [12] based on Real-time PCR technologies. In consideration of an inhomogeneous genotype concerning the *KRAS* mutation status of the tumor cells, more sensitive detection methods are required. If the tumor has a small content of *KRAS* mutated cells, not detectable by DNA sequencing, anti-EGFR therapy probably fail to have an anti-proliferating effect on these cells. 

The SNaPshot analysis is regarded as more reliable in comparison to common DNA sequencing [8] and able to detect tiny allele amounts of mutated *KRAS*. The aim of this study is to test the SNaPshot method in comparison to DNA sequencing for screening of *KRAS* mutation. 

## 2. Materials and Methods

### 2.1. Tumor Tissue Samples and DNA Extraction

Our study was carried out using tumor tissue samples from different sources: normal diagnostic cases of NSCLC (*n* = 41, 33 surgically resected specimens and 8 biopsy specimens) and colorectal carcinoma (*n* = 20, 18 surgically resected specimens and 2 biopsy specimens), archived pancreatic carcinomas (*n* = 19, 8 surgically resected specimens and 11 biopsy specimens), and FFPE (formalin-fixed paraffin-embedded) material of colorectal cancer specimens on slides from an interlaboratory comparison of *KRAS* mutation screening in spring 2008, organised by the German Society of Pathology. All specimens were made anonymous and examined by a pathologist, who choose a tumor-area with at least 70% of vital tumor cells for DNA-isolation. Weichert et al. [13] mentioned that specimens habroring fewer than 10% tumor cells showed lower mutation rates regardless of the method used.

The tumor tissue was fixed on slides (3 *μ*m thick), deparaffinated, and the tissue material was microdissected for DNA extraction using the QIAamp DNA Micro Kit (Qiagen, Hilden, Germany). Tissue material from the interlaboratory comparison on slides was identified by microscopy as tumor and normal tissue and DNA was extracted from both tissue samples (*n* = 20).

### 2.2. Amplification Step before SNaPshot and Sequencing Analysis

After extraction of the genomic DNA from the samples, the *KRAS* gene exon 2 was amplified by PCR (with HotStarTaq DNA Polymerase, Qiagen, Hilden, Germany) with the following primer set: 5′-AAGGCCTGCTGAAAATGACTG-3′ and 5′-CAAAGAATGGTCCTGCACCAG-3′ [8]. After checking the amplification product on an agarose gel, the PCR reaction was purified with the MinElute PCR Purification Kit (Qiagen, Hilden, Germany) and used for sequencing and the SNaPshot reaction.

Each PCR reaction had a 25 *μ*L volume and contained: the above-mentioned primer set with a concentration of 400 nM, 1  ×  PCR buffer, 2.5 mM MgCl_2_, 0.1 *μ*g/*μ*L BSA, 5 units of the polymerase, 200 *μ*M of each deoxynucleotide, and 100 ng of genomic DNA. The PCR reaction ran with the following program: 95°C initial denaturation for activating the HotStarTaq DNA Polymerase for 15 minutes, step 2 denaturation at 94°C for 30 seconds, step 3 annealing of the primer to the template at 55°C for 30 seconds, and step 4 30 seconds at 72°C for primer extension. Steps 2 to 4 were repeated 45 times followed by a final extension step at 72°C for 10 minutes, then the PCR reaction was cooled down to 8°C. One PCR preparation is used for sequencing reaction and the SNaPshot analysis.

### 2.3. DNA Sequencing

The purified DNA was used as a template for the sequencing reaction with the BigDye Terminatorv1.1 Cycle Sequencing Kit (Applied Biosystems, Forster City, CA). The sequencing reaction was performed with the 3′* kRAS* primer (5′-CAAAGAATGGTCCTGCACCAG-3′) with the following program on the thermocycler: 1 minute for an initial denaturation of the DNA at 96°C, followed by 25 cycles of a 10-second denaturation at 96°C, annealing of the primer at 50°C for 5 seconds, and the extension step at 60°C for 4 minutes. After this program, the samples were stored at 4°C.

**Figure 1:**
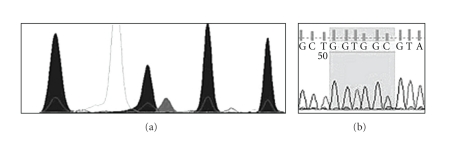
The electropherograms of the SNaPshot analysis (a) and the DNA sequencing (b) of the same tumor tissue sample. The mutation of codon 12 position 2 guanine to adenine transition is definitely visible in the SNaPshot analysis. In the sequencing analysis, the adenine-peak is hardly distinguishable from the background.

The capillary electrophoresis was performed on the 3130 Genetic Analyzer, and the obtained electropherogram (Figure 1(b)) was analysed with finchTV (Geospiza, Seattle, WA, USA) software (receivable online).

### 2.4. SNaPshot

The primers used for SNaPshot were implemented accordingly to the algorithm of Di Fiore et al. [8]: codon 12 position 1; 5′-AACTTGTGGTAGTTGGAGCT-3′, codon 12 position 2; 5′-GATCGTACTTGTGGTAGTTGGAGCTG-3′, codon 13 position 1; 5′-GATCGATCGATCTTGTGGTAGTTGGAGCTGGT-3′, codon 13 position 2; 5′-GATCGATCGATCGATCGATGTGGTAGTTGGAGCTGGTG-3′. These primers anneal one nucleotide position before the potential mutated base on the template DNA and in the course of polymerization with the SNaPshot Multiplex Kit (Applied Biosystems, Forster City, CA, USA), only this nucleotide is added as di-deoxy nucleotide to the primer sequence. A further polymerization is excluded, because of the lacking 3′-hydroxyl group on their nucleotide deoxyribose.

The SNaPshot reaction took place in a 10 *μ*L volume with 1 *μ*L PCR product, 5 *μ*L SNaPshot Multiplex Master Mix, and a 2 *μ*M concentration of each primer. The program on the thermocycler was 25 cycles of denaturation at 96°C for 10 seconds, primer-annealing at 50°C for 5 seconds, and an extension step at 60°C for 30 seconds then cooling down to 4°C. After the SNaPshot reaction was performed, a purification step was necessary to eliminate interfering-free nucleotides. Adding 1 *μ*L shrimp alkaline phosphatase (1 U/*μ*L) (NEB, Ipswich, MA) and incubating for 1 hour at 37°C, followed by a denaturation step at 75°C for 15 min, clean up the SNaPshot reaction for capillary electrophoresis on the 3130 Genetic Analyzer (Applied Biosystems, Forster City, CA). 

For capillary electrophoresis an internal DNA sizing ladder was used, the GeneScan-LIZ 120 Size Standard (Applied Biosystems, Forster City, CA). No further analysis program is necessary; the relevant data can be read directly from the electropherogram [8] (e.g., Figure 1(a)).

### 2.5. Statistical Analysis

The sensitivity and the specificity of SNaPshot analysis in *KRAS* mutation screening in comparison to common DNA sequencing was tested. The DNA sequencing was assumed as standard method with a specificity and sensitivity of 100%.

## 3. Results

In 45 (45%) out of 100 analysed tissue samples, a *KRAS* mutation in codon 12 or 13 were detected. 40 (88.9%) of these mutated *KRAS* genes were discovered by DNA sequencing and SNaPshot, a further five mutations (11.1%) were only detectable by SNaPshot. This results in a sensitivity of 100% with a confidence interval of 95% [91.2% to 100%] and a specificity of 91.7% with a confidence interval of 95% [81.6% to 97.2%], respectively.

Figure 1 shows an example of a mutation detection by SNaPshot but not by sequencing. The analysed cancer specimens were dissected in NSCLC, colorectal carcinoma, and pancreatic carcinoma. For NSCLC, we detected in 15/41 (36.6%) *KRAS*-mutated cancer tissue samples, in 6/20 (30%) *KRAS* mutations in colorectal specimens, in 14/19 (73.7%) pancreatic tumor samples, and in 9/20 (45%) colorectal tumor samples from the interlaboratory comparison. 

**Figure 2:**
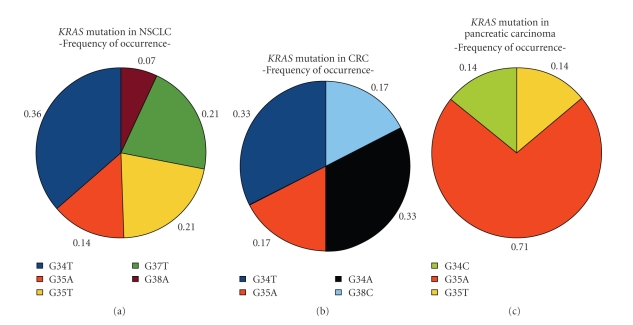
Delaminating of *KRAS* genotype of different tissue specimen. This picture shows the percentage of mutation types found in the particular kind of cancer specimens. The legend under the picture explains the colours used for the different genotypes.

The type of mutations in *KRAS* exon 2 codons 12 and 13 can be grouped in transitions and transversions. Transition mutations of *KRAS* exon 2 codons 12 and 13 occur in the majority (71.4%) of pancreatic carcinoma. NSCLC *KRAS* mutations mainly occur as transversions (78.6%) and *KRAS* mutations of colorectal carcinomas are evenly distributed as transition and transversion. In the NSCLC samples, G37T, G38A, G35T G34T, and G35A occur. The colorectal specimens show G34A, G38C, G35A, and G34T mutations. The pancreatic carcinomas show three different types of *KRAS* mutations: G35A, G34C, and G35T. The kind of *KRAS* mutations found in the tissue samples are explicitly listed in Figure 2 with the percentage of the total mutation number.

## 4. Discussion

The given percentage of *KRAS* mutation in NSCLC is 21–43% [4, 14] and 33% [15] for colorectal cancer and for pancreatic carcinomas 75–82.4% [15, 16]. *KRAS* mutation frequency in our examinations showed 36.6% for NSCLC and 30% for colorectal cancer. 73.7% of mutated *KRAS* genes in pancreatic carcinoma is in accordance with previously described data [15]. 

With regard to the present prerequisite of *KRAS* mutation screening before a therapy with Panitumumab and Cetuximab, *KRAS* is a powerful molecular marker in cancer diagnostics [1, 5]. The molecular cancer diagnostic is on the way to afford a more individualised therapy for these patients. This aim makes a molecular screening of the tumor necessary, which reveals the cancer characteristics correlated to the response to a chemotherapy.

Several studies indicate greater benefit from a Cetuximab therapy for EGFR expressing metastatic colorectal cancer patients with *KRAS* wild type [5–7]. Cetuximab in the first-line treatment combined with Oxaliplatin, Leucovorin, and Fluorouracil increases the overall response rate in patients with metastatic colorectal cancer [17].

Next to the important prediction of drug response for metastatic colorectal carcinomas, *KRAS* mutations may also be decisive for drug decision in NSCLC in regard to Erlotinib or Gefitinib, because these kinase inhibitors seem to be ineffective in carcinomas with *KRAS* mutation [12, 16, 17]. Eberhard et al. [14] showed a shorter median time to progression and survival in patients treated by Erlotinib and Cisplatin in case of mutated *KRAS *compared to patients receiving Cisplatin alone. *KRAS* does not seem to be a predictive factor [15] in a study with Gefitinib and chemotherapy for advanced colorectal cancer.

Particularly with regard to the expanding field of *KRAS* mutation status detection, it is necessary to determine the allele status in a reliable way. Therefore, knowledge of the *KRAS* gene mutation status is essential before the decision is made to treat a cancer patient using EGFR-targeted Panitumumab and Cetuximab therapy. Also considering the enormous costs and side effects of the therapy, the *KRAS* gene mutation analysis is necessary.

*KRAS* mutation can be screened in several ways: DNA sequencing, commercialised test systems by DNA-DNA hybridisation and SNaPshot analysis [8]. In this paper, we compare the common sequencing analysis and the SNaPshot, which is regarded to be more reliable [8]. Our results do reveal a better mutation detection, shown by the statistical analysis. The sensitivity reached 100% and the specificity 91.7%, implying a high probability of accuracy of the SNaPshot.

To ensure a positive mutation result and avoid false positive results, it is also recommended to run, next to a nucleic acid-free control sample (“water-control”), a *KRAS* wildtype control, with the known wildtype* KRAS* sequence.

The SNaPshot analysis shows a few advantages in addition to the enhanced reliability. It is less expensive and more rapid than the sequencing analysis (SNaPshot 11€ versus 19€ for DNA sequencing) and could be analysed without additional software, except the Genemapper program (Applied Biosystems, Forster City, CA, USA). Considering these qualities, the SNaPshot analysis could replace DNA sequencing in *KRAS* mutation screening.

The Therascreen: K-RAS Mutation Kit from Qiagen [12] uses a combination of ARMS-technology and Scorpions-primers, so it is possible to determine mutations of only 1% mutated allel in the background of wildtype genotype. But the kit could detect only 7 possible mutations types of the *KRAS* gene, but a whole of 12 mutations can occur in codons 12 and 13. In our analysis, we detected 2 mutations types, one in a colorectal carcinoma specimen, which would be detected as false negative with the therascreen kit.

The *KRAS *mutations can be subdivided in transversions and transitions. Both events lead to the exchange of the amino acid glycine to another amino acid in the protein. A transition is a replacement of one purine base to the other or one pyrimidine base to another, and a transversion is a replacement from a purine base to a pyrimidine base or vice versa. The occurrence of transition and transversion is not evenly distributed in these three different kinds of tumor tissue. Transversions appear more frequently in NSCLC and transitions more frequently in pancreatic carcinoma. Both mutation types occur evenly in colorectal carcinoma. An unequal frequency of occurrence of mutation types suggests different conditions in these three kinds of tumor. For a transition, just the addition or removal of an amino group is necessary. For a transversion, the base has to be removed and the new base has to be added. Riely et al. showed transition of the *KRAS* gene in lung cancer occurring more frequent in never smokers than in former or current smokers. This could imply that transversion mutations are associated with smoking [18].

For protocol of the *KRAS* detection only by SNaPshot, is it feasible to shorten the purification step of the PCR and use only a SAP/EXO degradation of interfering nucleic acids. Depending on the size of the PCR product, the SNaPshot is expandable to newly identified SNPs, simply by adding a suitable primer and PCR product to the reaction. This is a less expensive method to analyse SNPs in other genomic regions by just adding primer for the PCR and the SNaPshot reaction. However, the data cannot be reanalysed for unconsidered sequences. The SNaPshot has to be repeated with additional primers.

Tiny proportions of mutated *KRAS* alleles can easily be screened by the above-mentioned methods. In event of *KRAS* mutation, therapy with monoclonal antibodies Cetuximab and Panitumumab should be withdrawn [5, 11]. Tiny allele amounts of mutated *KRAS* suggest a majority of wild type cells concerning the *KRAS* gene in the tumor. The lack of quantification of *KRAS* mutation alleles may deprive patients of a treatment, which could be targeted on tumor cells with intact *KRAS* gene. Therefore, *KRAS* mutation detection with SNaPshot could have a more enhanced role in response prediction of therapy depending on which response can be expected due to the amount of mutated *KRAS* allele in the tumor.

##  Conflict of Interests

The authors indicated no potential conflicts of interests.
